# Determinants of healthy orthorexia and orthorexia nervosa: the roles of food security, dietary behaviors, and obesogenic environment

**DOI:** 10.3389/fnut.2026.1919473

**Published:** 2026-09-25

**Authors:** Tevfik Koçak, Tuba Eda Arpa Zemzemoğlu, Tuğba Türkkan, Kenan Bülbül, Birsen Yilmaz

**Affiliations:** 1 Department of Nutrition and Dietetics, Faculty of Health Sciences, Gümüşhane University, Gümüşhane, Türkiye; 2 Department of Social Work, Faculty of Health Sciences, Gümüşhane University, Gümüşhane, Türkiye; 3 Ministry of National Education, İsmail Yıldırım Primary School, Trabzon, Türkiye; 4 Tata Institute of Fundamental Research, Hyderabad, India; 5 Advanced Research Unit on Metabolism, Development and Ageing (ARUMDA), Tata Institute of Fundamental Research, Hyderabad, India

**Keywords:** food insecurity, healthy orthorexia, obesogenic environment, orthorexia nervosa, sustainable and healthy eating behaviors, ultra-processed foods

## Abstract

Orthorexia has recently gained attention as a two-dimensional construct centered on a shared health orientation. Recent conceptual developments distinguish healthy orthorexia (HeOr), defined as a non-pathological interest in healthy eating, from orthorexia nervosa (OrNe), characterized by rigid and compulsive food control accompanied by psychological and social impairment. This cross-sectional study investigated the associations of HeOr and OrNe with sociodemographic variables, dietary behaviors, and obesogenic environmental factors, and aimed to identify shared and distinct determinants of these tendencies with 1,015 adults aged 18–65 years. Orthorexia tendencies were assessed using the Teruel Orthorexia Scale. Additional measures included the Sustainable and Healthy Eating Behaviors Scale, Short Screening Questionnaire of Highly Processed Food Consumption, Adult Food Security Survey Module, Food Label Reading Attitude Scale, Food Choice Questionnaire, and the Obesogenic Environment Assessment Scale. In the adjusted regression models, HeOr was positively associated with sustainable and healthy eating behaviors (*β* = 0.26), AFSS food-insecurity severity score (*β* = 0.11), food label reading attitudes (*β* = 0.07), health-related food choice motives (*β* = 0.09), natural-ingredient preference (*β* = 0.14), weight-control motives (*β* = 0.15), ethical food choice motives (*β* = 0.10), and obesogenic social determinants and effects (*β* = 0.09). Conversely, affordability-related motives (*β* = −0.08), emotional appeal (*β* = −0.15), familiarity (*β* = −0.10), and obesogenic cultural determinants/access to experts (*β* = −0.12) were negatively associated with HeOr. For OrNe, positive associations were observed with sustainable and healthy eating behaviors (*β* = 0.13), AFSS food-insecurity severity score (*β* = 0.17), mood-related food choice motives (*β* = 0.13), weight-control motives (*β* = 0.26), familiarity (*β* = 0.09), ethical food choice motives (*β* = 0.18), and obesogenic social determinants and effects (*β* = 0.30), whereas health-related food choice motives (*β* = −0.12), affordability-related motives (*β* = −0.08), and emotional appeal (*β* = −0.24) showed negative associations. These findings suggest that orthorectic tendencies are associated with individual health motivations as well as broader behavioral and environmental contexts; however, because of the cross-sectional design, these associations should be interpreted as correlational and hypothesis-generating rather than causal.

## 1 Introduction

Recent literature suggests that there is a growing interest in healthy eating, although this occurs alongside increase in consumption of ultra-processed foods, the line between optimal nutrition and unhealthy, potentially obsessive eating behaviors remains conceptually and clinically unclear (1). Orthorexia nervosa (OrNe) is a rigid, distressing, and functionally disruptive obsession with “correct” or “pure” eating, while healthy orthorexia (HeOr) describes a non-pathological interest in food and nutritional quality without impairment (2). A consensus on the diagnosis of OrNe was achieved by a team of experts in 2022 (3). Failing to differentiate between health-focused eating and disorders might result in optimal health measures that are ineffectual and insufficient clinical definitions of these compulsive behaviors (4). Recent reviews highlight that cases of orthorexia are still not recognized in diagnostic systems and are difficult to define due to their overlap with eating disorders, obsessive-compulsive traits, anxiety, and socially valued “clean eating” norms (5, 6). The Teruel Orthorexia Scale (TOS) is particularly valuable in this context because it addresses orthorexia in two distinct dimensions: HeOr, reflecting a more deliberate interest in healthy eating, and OrNe, reflecting an excessive obsession with healthy eating associated with emotional, cognitive, and social impairment (7). The initial study (7) and subsequent validation studies validated this two-factor model, and current research continues to show significant variations in HeOr and OrNe’s psychological and diet-related associations (8, 9). Since orthorexia overlaps with eating disorder symptoms, obsessive-compulsive behaviors, anxiety, and culturally reinforced “clean eating” norms. Both dimensions share a common orientation toward healthy eating; therefore, positive correlations between them are theoretically plausible. HeOr reflects a flexible and non-impairing interest in healthy eating, whereas OrNe reflects a rigid, distressing, and potentially impairing preoccupation with dietary purity, correctness, and control. The scale developers tested whether the two TOS dimensions potentially associated with related psychopathological characteristics differently. OrNe was more consistently associated with eating disorder symptoms, obsessive-compulsive symptomatology, emotional distress, perfectionism, and impairment-related traits in the original and subsequent validation studies, while HeOr had weaker, nonexistent, or inverse associations with psychopathology. A person may show strong interest in healthy eating and still differ substantially in whether that interest remains adaptive or becomes compulsive, emotionally burdensome, and socially restrictive. The TOS’s psychometric significance resides in its ability to measure healthy eating interest and distinguish a flexible, non-harmful health orientation from a rigid and problematic obsession with dietary purity. This distinction is significant for this study because it permits HeOr and OrNe to be evaluated as linked but clinically and psychologically separate variables.

While many studies on orthorexia tendencies focus on individual-level psychological constructs such as perfectionism, obsessive-compulsive traits, anxiety, and disordered eating patterns, behaviors related to “healthy eating” are shaped not only by beliefs and traits, but also by nutritional knowledge, food security, and social norms (5, 10, 11). Sustainable (refers to dietary patterns that have low environmental impact while supporting nutritional adequacy, cultural acceptability, and long-term food system stability) and healthy eating may lead some people to seek ideal dietary quality, but it might reinforce moral or inflexible dietary patterns in others, making sustainability-related eating behavior an important but understudied interaction with orthorexia (2). Orthorexia and food insecurity are structurally important but insufficiently integrated issues, and this phenomenon may be linked to eating disorders through external dietary restrictions (12). Food insecurity, characterized by unreliable access to adequate and nutritious food, has been associated with disordered eating behaviors. A recent systematic review reported higher odds of binge eating among food-insecure individuals (13). Whether OrNe or HeOr is more strongly linked to food insecurity is still a question in the early stages of research on eating disorders and food insecurity (14). Although individuals with HeOr and OrNe may report similar types of food choices (e.g., preference for ‘healthy’ foods), their underlying motivations differ. HeOr is typically associated with health-oriented and natural food motivations, whereas OrNe is more strongly linked to rigid control, weight concerns, and dietary imbalance. The divergence is even more important when evaluating food label reading attitudes and food decision-making motivations. Food labels can reduce orthorexia by encouraging healthy eating and appropriate diets (15).

Taken together, these perspectives suggest that orthorectic tendencies are influenced by an interplay of psychological, behavioral, and environmental factors. However, existing research has not sufficiently integrated these domains, particularly in relation to food security, sustainable eating behaviors, and perceived obesogenic environments. Therefore, the present study aims to investigate the determinants of HeOr and OrNe by examining the roles of food security, dietary behaviors (including sustainable eating practices, ultra-processed food consumption, and food label reading), and perceived obesogenic environments. By integrating environmental, behavioral, and sociodemographic factors, this study seeks to provide a more comprehensive understanding of how structural food conditions may shape orthorectic tendencies. Because HeOr and OrNe are expected to be correlated, the present study does not treat them as opposite ends of a simple continuum. Instead, they are examined as related but distinct outcomes. This approach allows the analysis to identify correlates that are shared by both dimensions and correlates that are more specific to the maladaptive, impairment-related component of orthorexia. Additionally, this study tests the following hypotheses: H1: HeOr will be positively associated with food security, sustainable eating behaviors, and health-oriented dietary motivations. H2: OrNe will be associated with factors that enable dietary control (e.g., food security, structured food environments), reflecting rigid and compulsive eating patterns. H3: Sustainable eating behaviors will show positive associations with both HeOr and OrNe, but through distinct motivational pathways. H4: Different dimensions of the obesogenic environment will show distinct associations with orthorectic tendencies, with social environmental pressures positively associated, and cultural and economic factors showing differential or inverse relationships.

## 2 Method

### 2.1 Participants and setting

The study’s participant group consisted of 1,015 individuals, including 420 men (41.4%) and 595 women (58.6%), aged 18 to 65 years, with a mean age of 36.4 ± 13.30. For descriptive subgroup analyses, participants were classified into operational birth-cohort categories as follows: Generation X, born between 1965 and 1979; Generation Y, born between 1980 and 1999; and Generation Z, born in 2000 and later, within the eligible adult age range of the study. Accordingly, 34.0% of participants (*n* = 345) were classified as Generation X, 35.7% (*n* = 362) as Generation Y, and 30.3% (*n* = 308) as Generation Z. Ethical approval for the research was obtained from the Gümüşhane University Rectorate Scientific Research and Publication Ethics Committee on June 25, 2025, under document number E-95674917-108.99-341018. Data was gathered via in-person interviews conducted from September 2025 to January 2026. Participants were chosen from community environments, public areas, university/community locations, outpatient/community healthcare establishments, workplaces, or other real-world sites across all seven regions of Türkiye utilizing a non-probability, face-to-face sampling technique. The selected sampling locations aimed to engage adults with varied sociodemographic and lifestyle characteristics, rather than to establish a probability-based nationwide sample. The exclusion criteria were as follows: being younger than 18 years or older than 65 years, not providing informed consent, declining to continue the interview, or submitting an incomplete questionnaire. Records that did not satisfy these eligibility or completion criteria were excluded before the final analytical dataset was constructed. In addition, after eligibility screening, statistical outlier procedures were applied as described in the Data analysis section. All procedures performed in this study were conducted in accordance with the principles of the Declaration of Helsinki. The sufficiency of the sample size was assessed by both *a priori* power analysis and published guidelines for multiple regression modeling. *A priori* power analysis was performed utilizing G*Power version 3.1.9.7, presuming a medium effect size (f^2^ = 0.15), α = 0.05, statistical power (1 − β) = 0.95, and 17 predictors. The analysis revealed that at least 208 individuals would be necessary. Furthermore, Green’s guideline for evaluating individual predictors in multiple regression [*N* ≥ 104 + m, where m is the number of predictors] indicated a requisite minimum sample size of 121 participants. The final analytical sample comprised 1,015 adults, significantly surpassing both standards. The substantial sample size improved statistical precision, diminished the risk of overfitting, augmented the stability of regression coefficients, and offered adequate power to identify small-to-moderate associations among orthorexia dimensions, food security, dietary behaviors, food choice motives, and obesogenic environmental factors.

### 2.2 Demographic data

Data was collected to get information regarding participants’ gender, generation, education, chronic illnesses, medicine use, snack frequency, special diets, supplement usage, monthly income, income-to-nutrition ratio, residential region, and physical activity levels.

### 2.3 Teruel orthorexia scale (TOS)

Teruel Orthorexia Scale developed by Barrada and Roncero (7) the scale has 17 items and use a four-point Likert scale to evaluate both maladaptive and adaptive beliefs and actions around healthy eating. The scale consists of two dimensions: HeOr and OrNe. Healthy orthorexia defines a tendency toward nutritious foods and dietary habits, whereas OrNe indicates the adverse social and mental outcomes of aiming for an idealized healthy eating pattern. The Turkish validity and reliability of the scale were established by Asarkaya and Arcan (16). Cronbach’s alpha internal consistency coefficients are 0.86 for the HeOr subscale and 0.81 for the OrNe subscale. The reliability coefficients established for this study are 0.86 for the HeOr subscale and 0.88 for the OrNe subscale.

### 2.4 Sustainable and healthy eating behaviors scale

The “Sustainable and Healthy Eating Behaviors Scale” developed by Żakowska-Biemans et al. (17) and subsequently validated for reliability in Turkish by Köksal et al. (18), was utilized. The Turkish adaptation of the scale comprises 7 categories and 32 Likert-type items: “Quality Labels (Regional and Organic)”, “Seasonal Foods and Avoiding Food Waste”, “Animal Health”, “Reducing Meat Consumption”, “Healthy and Balanced Nutrition”, “Local Food”, and “Low Fat.” Participants were instructed to assess the questions utilizing a scale of “never,” “very rarely,” “rarely,” “sometimes,” “frequently,” “very often,” and “always.” “Never” equates to 1 point, while “always” corresponds to 7 points. This study utilized the total score of the scale. The reliability coefficient for the entire scale in the Turkish adaption study was shown to be 0.91. The reliability coefficient for the complete scale was established at 0.89.

### 2.5 Short screening questionnaire of highly processed food consumption (sQ-HPF)

The Short Screening Questionnaire of Highly Processed Food Consumption (sQ-HPF), originally developed by Martinez-Perez et al. (19) to provide a practical screening tool for highly processed food consumption in epidemiological and clinical studies, was used in its validated by Erdoğan Gövez et al. (20) Turkish form. The original sQ-HPF included 14 highly processed food and beverage items; however, during the Turkish validation study, three items were removed because of low factor loadings, resulting in an 11-item Turkish version (20). Therefore, the present study used the validated Turkish sQ-HPF consisting of 11 items: full-fat dairy products, processed meats, fats/oils, sugary and artificially sweetened beverages, sweets, snacks, ready-to-eat products, refined cereals, sauces, additives, and fried foods. For each item, a “yes” response indicating consumption above the specified threshold was assigned 1 point. The total score ranged from 0 to 11, with higher scores indicating greater highly processed food consumption. In the Turkish validation study, the internal consistency coefficient was reported as 0.65. In the present study, Cronbach’s alpha was 0.67.

### 2.6 Adult food security survey module

This scale is a Turkish adaptation of the US Adult Food Security Survey Module, intended to evaluate people’ food security status. The measure comprises 10 items that assess challenges in food access and related behaviors/experiences during the past 12 months. The items address situations encountered by individuals trying to fulfill their nutritional requirements, including inadequate food budgets, meal skips or portion loss, and lack of access to appropriate and balanced nutrition (21). In the Turkish version, scoring is calculated by aggregating the replies to the scale items. This adaption utilizes a Likert scale for a substantial number of components, giving a total score between 0 and 48, with a cutoff point of 10 established by ROC analysis. A total score between 0 and 10 indicates food security, while a score between 11 and 48 shows food insecurity (21). Therefore, the AFSS total score was used as a continuous indication of food-insecurity severity; higher scores indicate greater food insecurity. AFSS regression coefficients that are positive are associated with food insecurity.

### 2.7 Food label reading attitude scale

This scale was developed by Sığırcı and Sarp (22) to evaluate individuals’ food label reading habits. The questionnaire is a five-point Likert scale (strongly disagree = 1, strongly agree = 5) consisting of a total of 20 statements. The lowest and highest scores individuals can obtain on the scale are 20–100. As part of the scale development study, they determined the Cronbach alpha internal consistency coefficient for the scale as 0.93. For our study, the Cronbach alpha value was calculated as 0.92.

### 2.8 The food choice questionnaire

The food choice questionnaire is a valid and accurate instrument that assesses the aspects individuals contemplate when selecting meals. Steptoe (23) identified nine primary aspects affecting the meal choice process through their investigations conducted to develop the questionnaire. The factors include: health, mood (positive and negative emotions), accessibility (ease of preparation and use), sensory characteristics, nutritional content, price, body weight management (low energy and fat content), familiarity, and ethical considerations (officially approved country, environmentally sustainable packaging) Dikmen et al. (24) shown that this questionnaire is a valid and reliable instrument for analyzing the factors affecting food choice behavior in Turkish society, with an internal consistency measured by Cronbach’s alpha of 0.90. The Cronbach’s alpha value for our study was established at 0.92.

### 2.9 Assessment of the obesogenic environment scale

Assessment Scale of Obesogenic Environment for Adults, created by Kesik and Sağlam (25) to assess the obesogenic environment in Turkey, comprises 32 items and is divided into four sub-dimensions: physical environment and opportunity-related factors, cultural determinants and access to experts, social determinants and their impacts, and economic determinants and their effects. The scale’s internal consistency coefficient was determined to be 0.81. The Cronbach’s alpha internal consistency coefficient for this study was determined to be 0.76.

### 2.10 Data analysis

Prior to doing advanced analysis on the data acquired for this research, the dataset was investigated for specific assumptions. Assumptions required for multiple linear regression were evaluated prior to analysis. In this context, assumptions concerning outliers, normal distribution, and linearity were examined (32). Participant flow was clarified before the final analytical dataset was constructed. First, 45 records were excluded because they did not satisfy the predefined eligibility or completion criteria specify the actual reasons from the dataset, such as age outside the target range, incomplete questionnaire, withdrawal, duplicate record, or other documented reason. Second, univariate outliers were assessed by converting continuous variables into z-scores, and 31 records with values outside the ±3 range were excluded (26). Third, multivariate outliers were evaluated using Mahalanobis distance (27), Cook’s D values, and 85 additional records were excluded (28, 29). After these sequential exclusions, the final analytical sample consisted of 1,015 adults. Following the assessment of the data for outliers, the kurtosis and skewness values were evaluated. Table 1 presents the mean, standard deviation, minimum-maximum range, and kurtosis-skewness values for the scales.

**Table 1:** Descriptive findings of the scales used in the research and relationships between scale variables.

Variables	Mean	SD	Minimum	Maximum	Kurtosis	Skewness
TOS HeOr	12.86	6.18	0.00	27.00	−0.70	−0.01
TOS OrNe	5.98	5.20	0.00	21.00	−0.54	0.59
SHEBS	4.00	0.84	1.65	6.16	−0.10	−0.12
UPF	6.53	2.54	0.00	11.00	−0.61	−0.13
AFSS	4.56	7.24	0.00	35.00	1.79	1.65
FLRAS	69.60	15.43	26.00	100.00	−0.42	−0.35
FCQH	2.90	0.66	1.00	4.00	−0.23	−0.04
FCQM	2.81	0.71	1.00	4.00	−0.60	
FCQA	3.04	0.67	1.00	4.00	−0.58	
FCQEA	3.19	0.64	1.25	4.00	−0.59	
FCQNI	2.93	0.76	1.00	4.00	−0.64	
FCQP	2.96	0.70	1.00	4.00	−0.38	
FCQWC	2.71	0.77	1.00	4.00	−0.51	
FCQF	2.92	0.70	1.00	4.00	−0.61	
FCQEP	2.65	0.82	1.00	4.00	−0.81	
OPE	39.27	14.08	10.00	70.00	−0.56	−0.08
OCDAE	40.07	12.24	10.00	67.00	−0.68	0.00
OSDE	26.65	9.86	8.00	50.00	−0.84	0.11
OEDE	14.23	7.43	4.00	28.00	−1.03	0.17

SD, Standard deviation; TOS, Teruel Orthorexia Scale; HeOr, Healthy Orthorexia; OrNe: Orthorexia Nervosa; SHEBS, Sustainable and Healthy Eating Behaviors Scale; UPF, Ultra-Processed Foods; AFSS, Adult Food Security Survey score, higher scores indicate greater food insecurity; FLRAS, Food Label Reading Attitude Scale; FCS, Food Choice Questionnaire; FCQH, Food Choice Questionnaire Health; FCQM, Food Choice Questionnaire Mood; FCQA, Food Choice Questionnaire Affordability; FCQEA, Food Choice Questionnaire Emotional Appeal; FCQNI, Food Choice Questionnaire Natural Ingredients; FCQP, Food Choice Questionnaire Price; FCQWC, Food Choice Questionnaire Weight Control; FCQF, Food Choice Questionnaire Familiarity; FCQEP, Food Choice Questionnaire Ethical Principle; OPE, Obesogenic Physical Environment; OCDAE, Obesogenic Cultural Determinants and Access to Experts; OSDE, Obesogenic Social Determinants and Effects; OEDE, Obesogenic Economic Determinants and Effects.

## 3 Results

These subgroup comparisons are exploratory, unadjusted descriptive analyzes. Effect-size estimates might improve the descriptive interpretation of these group differences, but they were not generated for the *t*-test and ANOVA comparisons. Subgroup findings should be regarded cautiously and not overemphasized as the study’s main conclusion. The manuscript focuses on adjusted regression models, where R^2^ and normalized *β* coefficients provide explained variance and independent cross-sectional relationships. The mean HeOr scores of the subjects exhibited no statistically significant differences according to gender, chronic diseases, medication usage, or monthly income level (*p* > 0.05). Significant disparities in participants’ nutritional levels were observed based on generation [*F*(2, 1,012) = 4.97, *p* < 0.01], educational attainment [*F*(3, 1,011) = 5.03, *p* < 0.01], frequency of snacks [*F*(3, 1,011) = 3.03, *p* = 0.03], dieting practices [*t*(1013) = 3.58, *p* < 0.001], supplement consumption [*t*(1013) = 4.47, *p* < 0.01], the ratio of monthly income to nutrition [*F*(3, 1,011) = 8.75, *p* < 0.01], geographical region in Turkey [*F*(6, 1,008) = 2.84, *p* < 0.01], and physical activity levels [*t*(1013) = 4.42, *p* < 0.01]. Millennials (13.54 ± 6.10) exhibited a higher average age than Gen Z (12.04 ± 5.79). Individuals with a university degree or higher (13.53 ± 5.84) surpassed those with primary education (11.66 ± 7.15) and secondary education (11.85 ± 5.75) in average age. Those consuming three or more snacks (14.74 ± 6.22) had a higher average age compared to individuals with no snacks (12.62 ± 6.00) and those with one snack (12.51 ± 6.02). Participants adhering to a regular diet (17.03 ± 5.37) had a higher average age than those who did not (12.74 ± 6.16). Regular supplement users (15.74 ± 6.11) had a higher average age than non-users (12.60 ± 6.12). Lastly, individuals allocating their entire monthly income to nutrition (17.07 ± 5.31) had a higher average age. The mean HeOr scores exhibited statistically significant differences among individuals who allocated 1/3 of their time (13.77 ± 6.76), another 1/3 (12.79 ± 5.86), and 1/2 (11.88 ± 6.00), as well as between residents of the Central Anatolia region (13.90 ± 5.80) and the Eastern Anatolia region (14.45 ± 5.80) compared to those in the Mediterranean region (11.49 ± 6.75). Furthermore, the participants who regularly got involved in physical activity (13.95 ± 6.11) demonstrated significantly different scores from those who did not engage in such activities (12.20 ± 6.12).

As further shown in Table 2, the mean OrNe scores did not differ significantly according to participants’ gender, generation, educational level, chronic disease status, medication use, supplement use, monthly income, geographical region, or physical activity status (*p* > 0.05). The mean OrNe scores exhibited significant differences based on the number of snacks [*F*(3,1,011) = 4.16, *p* < 0.01], dieting status [*t*(1013) = 4.10, *p* < 0.01], and the ratio of monthly income to food expenditures [*F*(3,1,011) = 11.73, *p* < 0.01]. It was determined that individuals consuming two snacks (5.70 ± 5.17) exhibited significantly lower mean OrNe scores compared to those who did not snack (6.79 ± 5.20), as well as those who consumed three or more snacks (7.21 ± 5.93). Furthermore, individuals not adhering to a regular diet (5.86 ± 5.14) had significantly lower scores than those who did (9.92 ± 5.66). Additionally, those allocating 1/4 (6.68 ± 5.01), 1/3 (6.01 ± 5.16), and 1/2 (5.01 ± 5.14) of their monthly income to nutrition had significantly lower mean OrNe scores than those who spent their entire income on nutrition (10.28 ± 4.85).

**Table 2:** Sociodemographic, characteristic, nutritional and lifestyle findings of the participants.

Variables	Category	*n* (%)	Healthy orthorexia		Orthorexia nervosa	
			Mean ± SD	*p*	Mean ± SD	*p*
Sex	Female	595 (58.6)	12.99 ± 6.16	0.41^a^	6.03 ± 5.15	0.71 ^a^
	Male	420 (41.4)	12.67 ± 6.20		5.90 ± 5.26	
Generation	^1^X (1965–79)	345 (34.0)	12.87 ± 6.51	0.01^b^ 3 < 2	5.81 ± 5.36	0.19^b^
	^2^Y (1980–99)	362 (35.7)	13.54 ± 6.10		6.37 ± 5.32	
	^3^ Z (2000 and later)	308 (30.3)	12.04 ± 5.79		5.70 ± 4.84	
Education	^1^Primary	160 (15.8)	11.66 ± 7.15	0.01 ^b^ 4 > 1, 3	4.97 ± 5.86	0.06^b^
	^2^Secondary	119 (11.7)	11.85 ± 5.75		5.95 ± 5.14	
	^3^High school	247 (24.3)	12.78 ± 6.19		6.13 ± 5.07	
	^4^University and above	489 (48.2)	13.53 ± 5.84		6.23 ± 5.01	
Chronic disease	Yes	188 (18.5)	12.54 ± 6.48	0.44 ^a^	6.12 ± 5.39	0.67 ^a^
	No	827 (81.5)	12.93 ± 6.11		5.94 ± 5.15	
Medication use	Yes	147 (14.5)	13.04 ± 6.49	0.69 ^a^	6.36 ± 5.24	0.34 ^a^
	No	868 (85.5)	12.83 ± 6.12		5.91 ± 5.19	
Snack frequency	^1^None	183 (18.0)	12.62 ± 6.00	0.03 ^b^ 4 > 1, 2	6.79 ± 5.20	0.01 ^b^ 3 < 1, 4
	^2^1 Snack	381 (37.5)	12.51 ± 6.02		5.70 ± 5.17	
	^3^2 Snacks	369 (36.4)	12.91 ± 6.36		5.58 ± 4.97	
	^4^3 Snacks and more	82 (8.1)	14.74 ± 6.22		7.21 ± 5.93	
Being on a diet	Yes	28 (2.8)	17.03 ± 5.37	0.00 ^a^	9.92 ± 5.66	0.00 ^a^
	No	987 (97.2)	12.74 ± 6.16		5.86 ± 5.14	
Supplement use	Yes	83 (8.2)	15.74 ± 6.11	0.00 ^a^	6.44 ± 5.35	0.40 ^a^
	No	932 (91.8)	12.60 ± 6.12		5.93 ± 5.18	
Monthly income covers expenses	Above	292 (28.8)	13.50 ± 5.91	0.06 ^b^	5.82 ± 5.16	0.83 ^b^
	Equal	462 (45.5)	12.78 ± 6.24		6.02 ± 5.23	
	Below	261 (25.7)	12.28 ± 6.32		6.07 ± 5.19	
Percentage of monthly income spent on food	^1^1/4	233 (23.0)	13.77 ± 6.76	0.00 ^b^ 4 > 1,2,3	6.68 ± 5.01	0.00 ^b^ 4 > 1,2,3
	^2^1/3	448 (44.1)	12.79 ± 5.86		6.01 ± 5.16	
	^3^1/2	306 (30.1)	11.88 ± 6.00		5.01 ± 5.14	
	^4^1/1	28 (2.8)	17.07 ± 5.31		10.28 ± 4.85	
The region of lives in Türkiye	^1^Marmara.	125 (12.3)	13.24 ± 6.00	0.01 ^a^ 3 > 4 4 < 6	6.08 ± 4.57	0.32 ^a^
	^2^Aegean	57 (5.6)	13.17 ± 5.16		7.47 ± 4.78	
	^3^Central Anatolia	125 (12.3)	13.90 ± 5.80		5.76 ± 4.40	
	^4^Mediterranean	153 (15.1)	11.49 ± 6.75		5.37 ± 5.51	
	^5^Black Sea	413 (40.7)	12.77 ± 6.29		6.03 ± 5.47	
	^6^Eastern Anatolia	64 (6.3)	14.45 ± 5.80		7.23 ± 5.56	
	^7^Southeastern Anatolia	78 (7.7)	12.20 ± 5.75		4.97 ± 4.88	
Physical activity	Regular	382 (37.6)	13.95 ± 6.11	0.00 ^a^	6.19 ± 4.94	0.31 ^a^
	Irregular	633 (62.4)	12.20 ± 6.12		5.85 ± 5.34	

^a^Independent-samples *t*-test, ^b^one-way ANOVA; SD, standard deviation.

Table 1 demonstrates that all scales have kurtosis and skewness values within the ±2 range, supporting the assumption of normal distribution (30). Durbin-Watson coefficient was used to test regression analysis’ autocorrelation assumption. This number was 1.89 for HeOr and 2.00 for OrNe. Since the Durbin-Watson value is 1–3 (31), there is no autocorrelation and the error factors are independent. Variance Inflating Factor (VIF) and Tolerance values were used to test multicollinearity. The study met the assumption of multicollinearity because the correlation coefficients were less than 0.90, VIF values were less than 5, and tolerance values were greater than.20 (HeOr = 0.48–0.93; OrNe = 0.48–0.93) (32, 33). Pearson correlation analysis was used to investigate variable associations when all assumptions were met. To explore HeOr and OrNe score differences among sociodemographic, nutritional, and lifestyle subgroups, independent-samples *t*-tests and one-way ANOVA analyses were employed. These analyses were not the study’s main inference framework. Because these subgroup comparisons were exploratory and unadjusted, they were used to describe potential group-level patterns rather than to establish confirmatory subgroup effects. Separate effect-size estimates for these *t*-test and ANOVA comparisons were not calculated; therefore, interpretation of these subgroup findings was based on descriptive patterns and *p*-values with appropriate caution. In contrast, the primary adjusted analyses were the regression models, for which R^2^ and standardized *β* coefficients were reported. Multiple linear regression analyses were then used to test the effects of sustainable and healthy eating behaviors, ultra-processed food consumption, AFSS food-insecurity severity score, food label reading attitudes, food choice status, and obesogenic environment (physical environment, cultural determinants and access to experts, social determinants and their effects, economic determinants and their effects) on HeOr and OrNe.

The correlation matrix was also interpreted as an exploratory map of bivariate associations; therefore, the pattern and coherence of correlations were emphasized rather than individual significance tests alone. According to Figure 1, multiple statistically significant relationships were observed between orthorexia dimensions, food choice motives, obesogenic eating behaviors, and lifestyle-related variables. Healthy orthorexia (HeOr) correlated positively with orthorexia nervosa (OrNe) (*r* = 0.64, *p* < 0.01) demonstrating a partial overlap between health-oriented eating patterns and OrNe. This moderate-to-strong positive association indicates that HeOr and OrNe share a common health-eating orientation, but it does not mean that the two constructs are identical; rather, it supports interpreting the subsequent regression models as separate analyses of two correlated orthorexia dimensions. Also, HeOr was positively correlated with SHEBS (*r* = 0.49, *p* < 0.01), FLRAS (*r* = 0.41, *p* < 0.01), FCQH (*r* = 0.45, *p* < 0.01), FCQNI (*r* = 0.48, *p* < 0.01), and FCQWC (*r* = 0.49, *p* < 0.01). In contrast, HeOr was significantly and negatively associated with UPF intake (*r* = −0.17, *p* < 0.01) and OCDAE scores (*r* = −0.45, *p* < 0.01). These results show that greater health conscious orthorexic tendencies were associated with greater health-conscious food choices and less engagement with obesogenic dietary environments.

**Figure 1:**
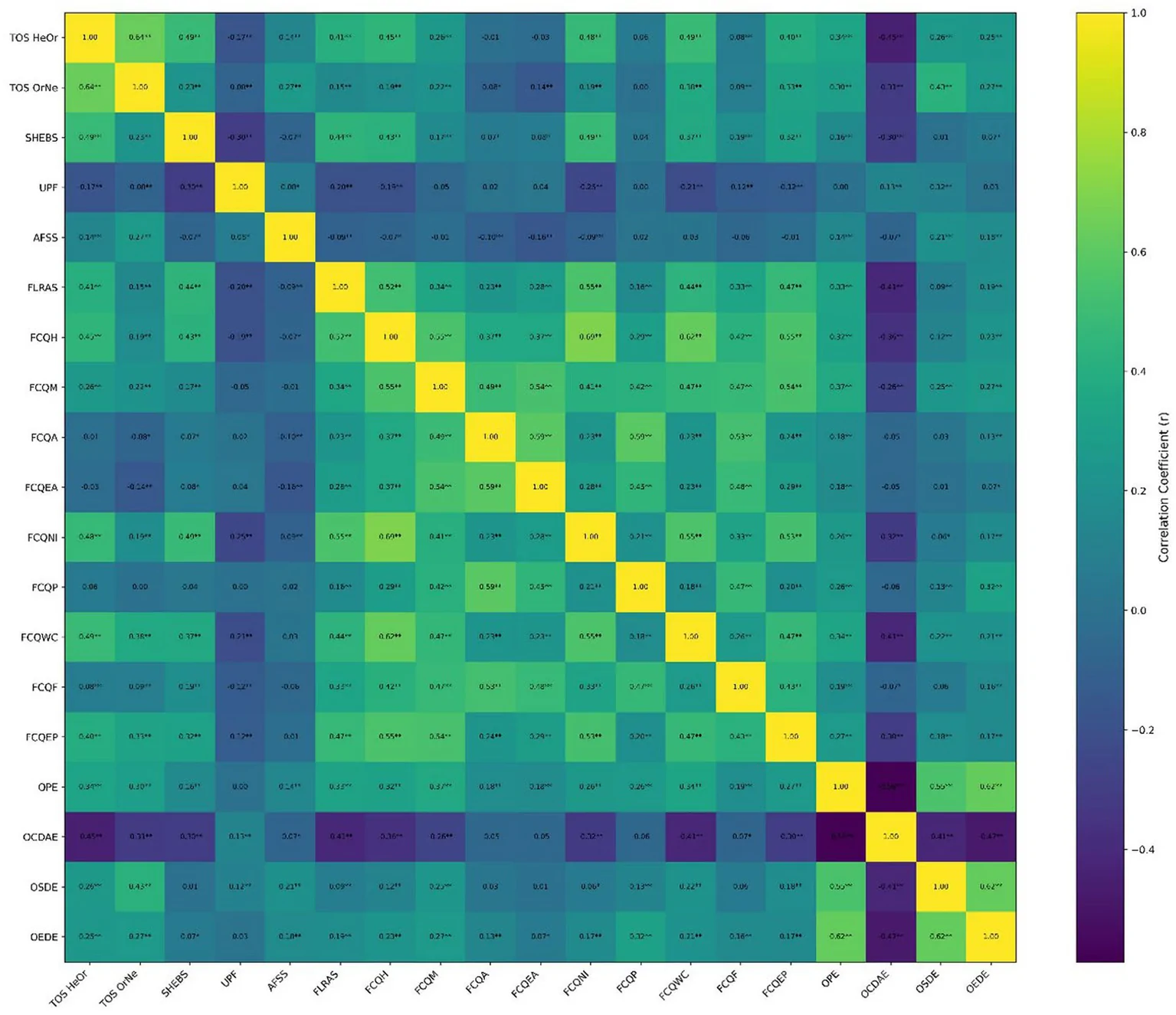
Relationships between scale variables used in the research. TOS, Teruel Orthorexia Scale; HeOr, Healthy Orthorexia; OrNe, Orthorexia Nervosa; SHEBS, Sustainable and Healthy Eating Behaviors Scale; UPF, Ultra-Processed Foods; AFSS, Adult Food Security Survey score; higher scores indicate greater food insecurity; FLRAS, Food Label Reading Attitude Scale; FCS, Food Choice Questionnaire; FCQH, Food Choice Questionnaire Health; FCQM, Food Choice Questionnaire Mood; FCQA, Food Choice Questionnaire Affordability; FCQEA, Food Choice Questionnaire Emotional Appeal; FCQNI, Food Choice Questionnaire Natural Ingredients; FCQP, Food Choice Questionnaire Price; FCQF, Food Choice Questionnaire Familiarity; FCQEP, Food Choice Questionnaire Ethical Principle; OPE, Obesogenic Physical Environment; OCDAE, Obesogenic Cultural Determinants and Access to Experts; OSDE, Obesogenic Social Determinants and Effects; OEDE, Obesogenic Economic Determinants and Effects.

OrNe was positively linked with some food choice dimensions: AFSS (*r* = 0.27, *p* < 0.01), FCQWC (*r* = 0.38, *p* < 0.01), FCQEP (*r* = 0.33, *p* < 0.01), OPE (*r* = 0.30, *p* < 0.01) and OSDE (*r* = 0.43, *p* < 0.01). In contrast, negative relationships were found between OrNe and UPF consumption (*r* = −0.08, *p* < 0.01), FCQEA (*r* = −0.14, *p* < 0.01), and OCDAE (*r* = −0.31, *p* < 0.01). These findings suggest that orthorexia nervosa may co-occur with both restrictive dietary patterns and increased sensitivity to specific food-related environmental signals.

The SHEBS scores were positively and significantly correlated with FLRAS (*r* = 0.44, *p* < 0.01), FCQH (*r* = 0.43, *p* < 0.01), FCQNI (*r* = 0.49, *p* < 0.01), FCQWC (*r* = 0.37, *p* < 0.01), and FCQEP (*r* = 0.32, *p* < 0.01), and negatively correlated with UPF intake (*r* = −0.30, *p* < 0.01) and OCDAE (*r* = −0.30, *p* < 0.01). The findings imply that sustainable healthy eating behaviors are associated with higher nutritional awareness and reduced obesogenic exposure.

Several healthy eating related characteristics were negatively connected to UPF consumption like FLRAS (*r* = −0.20, *p* < 0.01), FCQH (*r* = −0.19, *p* < 0.01), FCQNI (*r* = −0.25, *p* < 0.01), FCQWC (*r* = −0.21, *p* < 0.01) and FCQF (*r* = −0.12, *p* < 0.01). However, positive relationships were observed between UPF consumption and AFSS (*r* = 0.08, *p* < 0.05), OCDAE (*r* = 0.13, *p* < 0.01) and OSDE (*r* = 0.12, *p* < 0.01). This pattern suggests that increased intake of ultra-processed foods may be associated with more obesogenic and less health-oriented diets.

Strong positive intercorrelations were found between food choice dimensions. FCQH was significantly correlated with FCQNI (*r* = 0.69, *p* < 0.01), FCQWC (*r* = 0.62, *p* < 0.01), FCQEP (*r* = 0.55, *p* < 0.01), and FCQM (*r* = 0.55, *p* < 0.01). The correlation between FCQA and FCQEA was moderate to strong (*r* = 0.59, *p* < 0.01), indicating substantial conceptual overlap between affordability-related and emotional-appeal food choice motives. These findings suggest that there is a large conceptual overlap across health-, nutrition-, and weight-control-related food choice reasons.

Regarding obesogenic environment-related variables, OPE was negatively associated with OCDAE (*r* = −0.59, *p* < 0.01) and positively associated with OSDE (*r* = 0.55, *p* < 0.01) and OEDE (*r* = 0.62, *p* < 0.01). Furthermore, OCDAE was inversely correlated with OSDE (*r* = −0.41, *p* < 0.01) and OEDE (*r* = −0.47, *p* < 0.01). Collectively, these findings suggest that healthier eating attitudes and behaviors are inversely correlated with obesogenic environmental characteristics and dietary exposures.

Multiple linear regression models for HeOr and OrNe were statistically significant. The HeOr model explained 48% of the variance, while the OrNe model explained 41% of the variance. Because Table 3 reports both unstandardized coefficients and standardized coefficients, unstandardized coefficients are presented as B, whereas standardized coefficients are interpreted as *β* to compare the relative strength of associations. TOS HeOr: *F*(17,997) = 55.10, *p* < 0.001; TOS OrNe: *F*(17,997) = 40.45, *p* < 0.001. In the HeOr model, sustainable and healthy eating behaviors (B = 1.90, *β* = 0.26, *t* = 9.26, *p* < 0.001), AFSS food-insecurity severity score (B = 0.09, *β* = 0.11, *t* = 4.59, *p* < 0.001), food label reading attitudes (B = 0.03, *β* = 0.07, *t* = 2.44, *p* = 0.01), health-related food choice motives (B = 0.87, *β* = 0.09, *t* = 2.46, *p* = 0.01), natural-ingredient preference (B = 1.10, *β* = 0.14, *t* = 3.86, *p* < 0.001), weight-control motives (B = 1.22, *β* = 0.15, *t* = 4.76, *p* < 0.001), ethical food choice motives (B = 0.73, *β* = 0.10, *t* = 3.04, *p* < 0.001), and obesogenic social determinants and effects (B = 0.06, *β* = 0.09, *t* = 2.78, *p* = 0.01) were positively associated with HeOr. *n* the OrNe model, sustainable and healthy eating behaviors (B = 0.82, *β* = 0.13, *t* = 4.43, *p* < 0.001), AFSS food-insecurity severity score (B = 0.12, *β* = 0.17, *t* = 6.55, *p* < 0.001), mood-related food choice motives (B = 0.92, *β* = 0.13, *t* = 3.42, *p* < 0.001), weight-control motives (B = 1.73, *β* = 0.26, *t* = 7.52, *p* < 0.001), familiarity (B = 0.65, *β* = 0.09, *t* = 2.65, *p* = 0.01), ethical food choice motives (B = 1.16, *β* = 0.18, *t* = 5.37, *p* < 0.001), and obesogenic social determinants and effects (B = 0.16, *β* = 0.30, *t* = 8.63, *p* < 0.001) were positively associated with OrNe. This pattern indicates that HeOr and OrNe share some health-oriented and food-control correlates, but OrNe is more strongly characterized by social obesogenic pressure and weight-control-related motives.

**Table 3:** Regression results to explain healthy orthorexia and orthorexia nervosa.

Model		Unstandardized coefficients		Standardized coefficients	*t*	Sig.	*R*	R^2^
		B	S. E.	Beta				
Healthy orthorexia (TOS)	(Constant)	−0.85	1.63		−0.52	0.60	0.70	0.48
	SHEBS	1.90	0.21	0.26	9.26	**< 0.001**		
	UPF	0.03	0.06	0.01	0.42	0.67		
	AFSS	0.09	0.02	0.11	4.59	**< 0.001**		
	FLRAS	0.03	0.01	0.07	2.44	**0.01**		
	FCQH	0.87	0.35	0.09	2.46	**0.01**		
	FCQM	0.39	0.30	0.04	1.30	0.19		
	FCQA	−0.70	0.31	−0.08	−2.27	**0.02**		
	FCQEA	−1.47	0.31	−0.15	−4.77	**< 0.001**		
	FCQNI	1.10	0.28	0.14	3.86	**< 0.001**		
	FCQP	0.42	0.27	0.05	1.54	0.12		
	FCQWC	1.22	0.26	0.15	4.76	**< 0.001**		
	FCQF	−0.84	0.27	−0.10	−3.10	**< 0.001**		
	FCQEP	0.73	0.24	0.10	3.04	**< 0.001**		
	OPE	0.01	0.02	0.03	0.93	0.35		
	OCDAE	−0.06	0.02	−0.12	−3.78	**< 0.001**		
	OSDE	0.06	0.02	0.09	2.78	**0.01**		
	OEDE	−0.01	0.03	−0.02	−0.48	0.63		
Orthorexia nervosa (TOS)	(Constant)	−1.16	1.48		−0.78	0.43	0.64	0.41
	SHEBS	0.82	0.19	0.13	4.43	**< 0.001**		
	UPF	−0.04	0.05	−0.02	−0.65	0.51		
	AFSS	0.12	0.02	0.17	6.55	**< 0.001**		
	FLRAS	−0.01	0.01	−0.04	−1.32	0.19		
	FCQH	−0.93	0.32	−0.12	−2.92	**< 0.001**		
	FCQM	0.92	0.27	0.13	3.42	**< 0.001**		
	FCQA	−0.65	0.28	−0.08	−2.34	**0.02**		
	FCQEA	−1.93	0.28	−0.24	−6.93	**< 0.001**		
	FCQNI	−0.10	0.26	−0.02	−0.40	0.69		
	FCQP	−0.13	0.25	−0.02	−0.52	0.60		
	FCQWC	1.73	0.23	0.26	7.52	**< 0.001**		
	FCQF	0.65	0.24	0.09	2.65	**0.01**		
	FCQEP	1.16	0.22	0.18	5.37	**< 0.001**		
	OPE	0.01	0.01	0.03	0.92	0.36		
	OCDAE	−0.01	0.01	−0.02	−0.58	0.56		
	OSDE	0.16	0.02	0.30	8.63	**< 0.001**		
	OEDE	−0.03	0.03	−0.05	−1.29	0.20		

TOS, Teruel Orthorexia Scale; HeOr, Healthy Orthorexia; OrNe, Orthorexia Nervosa; SHEBS, Sustainable and Healthy Eating Behaviors Scale; UPF, Ultra-Processed Foods; AFSS, Adult Food Security Survey score, higher scores indicate greater food insecurity; FLRAS, Food Label Reading Attitude Scale; FCS, Food Choice Questionnaire; FCQH, Food Choice Questionnaire Health; FCQM, Food Choice Questionnaire Mood; FCQA, Food Choice Questionnaire Affordability; FCQEA, Food Choice Questionnaire Emotional Appeal; FCQNI, Food Choice Questionnaire Natural Ingredients; FCQP, Food Choice Questionnaire Price; FCQWC, Food Choice Questionnaire Weight Control; FCQF, Food Choice Questionnaire Familiarity; FCQEP, Food Choice Questionnaire Ethical Principle; OPE, Obesogenic Physical Environment; OCDAE, Obesogenic Cultural Determinants and Access to Experts; OSDE, Obesogenic Social Determinants and Effects; OEDE, Obesogenic Economic Determinants and Effects. Bold values indicate statistical significance (*p* < 0.05).

## 4 Discussion

The aim of this study is to examine the relationship between HeOr and OrNe levels and sociodemographic variables, food security and eating behaviors, and obesogenic environmental factors, and to identify the common and differentiating determinants of these two constructs. The results revealed that orthorectic tendencies were consistent across several important demographic characteristics, although displayed considerable variations concerning generation, educational attainment, and specific lifestyle markers. No significant variations were observed in HeOr or OrNe scores concerning gender, chronic disease presence, medication usage, and monthly income level. Conversely, individuals from the Generation Y, those possessing a university degree or higher, those participating in regular physical activity, those adhering to dietary regimens, those utilizing nutritional supplements, and those dedicating a greater proportion of their income to nutrition exhibited elevated levels of HeOr. Orthorexia nervosa, although apparently independent of many demographic factors, was identified as being correlated with several behavioral indicators, including snack frequency, dietary habits, and the proportion of income spent to nutrition. Multiple regression analyses showed that the two orthorexia dimensions had partially overlapping but clearly distinct multivariable profiles (48% for HeOr, 41% for OrNe). HeOr was independently associated with sustainable and healthy eating behaviors, label-reading attitudes, health-oriented and natural-ingredient food motives, weight-control and ethical motives, AFSS food-insecurity severity, and obesogenic social determinants. OrNe, in contrast, showed its strongest independent association with obesogenic social determinants, followed by weight-control motives, emotional appeal in the negative direction, ethical motives, AFSS food-insecurity severity, sustainable eating behaviors, mood-related motives, and familiarity. Importantly, variables such as UPF consumption and economic obesogenic environment should not be interpreted as independent predictors in the adjusted models because their regression coefficients were not statistically significant. For analyzing these models and understanding the two TOS aspects at the individual level, the positive correlation between HeOr and OrNe is key. This does not mean that HeOr and OrNe are interchangeable or that they are opposing extremities of a continuum from “normal” to “nervotic” orthorexia. Instead, both aspects appear to have a health-oriented eating core but differ in psychological quality and functional consequence. Strong interest in healthy eating may stay flexible, non-impairing, and integrated into daily life, or it may become rigid, distressing, socially restricted, and compulsively regimented. Instead of classifying individuals as HeOr-type or OrNe-type, the regression models identify which cross-sectional correlates are more closely associated with adaptive vs. maladaptive health-oriented eating. HeOr was more associated with sustainable and health-oriented eating behaviors, label reading, natural-ingredient preference, and flexible health-oriented dietary engagement, while OrNe was more associated with obesogenic social determinants, weight-control motives, ethical food-choice concerns, food-insecurity severity, and lower emotional appeal. The data support a “shared core–divergent expression” interpretation: HeOr and OrNe share a desire for healthy eating, but they differ in rigidity, psychological burden, and social impairment. A literature study indicates a notable inconsistency in findings concerning the links between orthorectic tendencies and demographic characteristics. A study investigating orthorexic tendencies in two distinct cohorts of young adults revealed no consistent correlation between the risk of orthorexia and gender, educational attainment, geographic location, or profession (34). A study conducted in Turkey using a community sample and including 350 participants aged 18–65 also reported that, in line with the findings of this study, there was no significant difference between genders in terms of HeOr and OrNe scores (35). Similarly, a study of 814 participants aged 18–77 revealed no significant differences between women and men in terms of either HeOr or OrNe (36). In contrast, a study conducted with a relatively large sample of individuals aged 19–66 reported that gender was a strong predictor of orthorexia tendencies (37). Likewise, research on health condition and drug utilization reveals discrepancies in the literature. Contrary to the findings of this study, Arpa Zemzemoğlu et al. (38) indicated that persons with a specified health condition who consistently utilize medicine demonstrated elevated orthorectic tendencies (38). In contrast, in the study conducted by Karakus et al. (39) with university students, it was determined that orthorectic tendencies did not differ statistically significantly according to the chronic disease status variable. In contrast, a more recent study among university students revealed that monthly income did not exhibit a significant correlation with orthorexia nervosa (40). In conclusion, these conflicting and inconsistent results indicate that demographic variables may possess restricted predictive capacity in elucidating orthorectic tendencies; in contrast, the incorporation of psychological variables and individual processes in research models may offer greater explanatory power. Moreover, a significant amount of the differences noted in the literature may be attributed to the psychometric deficiencies of the employed assessment instruments. Numerous studies highlight that ORTO-15 and its derivatives, commonly utilized to evaluate orthorectic tendencies and validated for reliability in Türkiye by Arusoğlu et al. (37), exhibit insufficient diagnostic sensitivity to precisely and accurately assess orthorexia nervosa. This situation may result in mistaken positive outcomes in persons who do not exhibit the whole symptomatology of orthorexia nervosa (41). The utilization of psychometrically valid and multidimensional measurement instruments that distinguish between healthy and abnormal aspects of orthorexia is essential for a more consistent interpretation of the literature’s findings.

Healthy orthorexia and OrNE had different motivational and contextual characteristics despite a similar food and health focus, which is crucial. Both aspects were positively correlated with sustainable and healthy eating and food security, while weight-control goals, ethical food views, and perceived societal obesogenic influences predicted orthorexia nervosa the most. Healthy orthorexia was more linked to health-oriented, natural-content, and sustainability-related dietary choices. Recent studies indicate that orthorexia nervosa is a distinct phenomenon with rigid self-regulation, cognitive inflexibility, and moralized food beliefs, rather than just an intensified form of healthy eating motivation (11, 42, 43). Thus, psychological and motivational factors rather than food choices appear to distinguish healthy from OrNE.

In the present study, participants classified as Generation Y (1980–1999) had higher mean HeOr scores than those classified as Generation Z (2000 and later). No significant difference was observed in OrNe scores across the operational generation groups. This finding shows that intergenerational differences may be more closely associated with functional and adaptive attitudes toward healthy eating; in contrast, orthorexia nervosa may demonstrate a structure that is relatively independent of demographic factors such as age or generation. A review of the literature reveals inconsistencies in the findings regarding the relationship between age and orthorectic tendencies. Some studies conducted with adult samples have reported no significant associations between age and the risk of OrNe (34, 44, 45). Similarly, studies conducted in Turkey have also found that age is not a primary variable determining orthorectic tendencies (37, 46). In contrast, another study conducted in Poland by Dittfeld et al. (47) reported that the risk of OrNe decreased with age; orthorectic tendencies (health food fanaticism) were higher in younger individuals, and the highest rates were observed in the 19 years and under group. A study in Spain including 460 university students indicated that age exhibited a low-level positive connection with HeOr and a low-level negative correlation with OrNe (48). Conversely, in the research conducted by Barlow et al. (49), age was treated as a demographic control variable; nonetheless, it was noted that the associations between HeOr and OrNe and age did not exhibit a consistent or significant pattern across the various measurement instruments employed. Upon evaluating these studies collectively, it is posited that age and generational variations may correlate with HeOr to some degree; nonetheless, orthorexia nervosa appears to be more intricately associated with cognitive, affective, and psychological processes than with demographic characteristics. This situation highlights once again the importance of holistic and multidimensional approaches that focus on distinguishing between HeOr and OrNe, rather than focusing on single demographic variables such as age, in understanding orthorectic tendencies.

Analysis revealed that people possessing a university degree or higher exhibited markedly elevated HeOr score in comparison to those with only primary or secondary education. No substantial difference was observed in OrNe scores based on educational level. This outcome indicates that although educational attainment increases conscious attitudes toward healthy eating, it may not be directly associated with OrNe tendencies. Previous studies on this subject have produced inadequate information or conflicting outcomes. Research in Turkey showed individuals with postgraduate education exhibited diminished orthorectic tendencies relative to those with other educational qualifications (37). On the other hand, in a study by Peres et al. (50) to examine the relationships between individual characteristics and OrNe and HeOr in a sample of adults, education and income were not found to be significantly associated with either dimension of orthorexia. However, in a study conducted by Donini et al. (51) to propose a diagnostic recommendation for OrNe in a sample of 404 individuals, they reported a higher prevalence of OrNe among men and individuals with lower levels of education. In addition, there is research showing a relationship between educational level and maladaptive eating patterns, and that women with lower levels of education are more likely to suffer from eating disorders (52). However, most studies have been conducted with university students (53, 54) and have generally focused on orthorexic tendencies in groups with and without education in health fields such as medicine, nutrition, and dietetics (55). The findings of our current study indicate that the two components of orthorexia are grounded in distinct psychosocial dynamics. Although educational levels may serve a reinforcing function in the context of HeOr, the most critical determinants in the case of OrNe are individual vulnerability characteristics, like psychological conditions, cognitive anomalies, or challenges in emotional regulation. This distinction is also important for preventive and intervention-based studies.

Analyses indicated that individuals having three or more snacks daily showed substantially higher HeOr scores in comparison to those who consumed no snacks or only one snack per day. In contrast, people who consumed two snacks daily showed significantly lower OrNe scores than those who either abstained from snacking or consumed three or more snacks. This discovery indicates that snacking behavior may fulfill distinct roles concerning HeOr and OrNe tendencies. The literature lacks conclusive evidence concerning the correlation between snacking frequency and tendencies toward orthorexia. A cross-sectional study with 4,003 participants aged 18–45 indicated that ORTO-11 scores diminished with a higher frequency of snack consumption (56). In contrast, another study has shown that individuals with a higher predisposition to orthorexia nervosa consume more snacks. A study conducted with Polish university students, involving 1,120 participants, reported that students who consumed snacks most frequently were less likely to exhibit OrNe tendencies (57). Another study examining meal frequency and OrNe tendencies in adults found a negative but statistically negligible correlation between ORTO-11 scores and the number of main meals, snacks, and total meals (58). Similarly, a study conducted with women aged 20–40 found no significant difference in snack consumption between women with and without orthorexia tendencies (59). No research directly investigating the correlation between HeOr and snacking behavior has been identified in the literature. When the findings are together looked at, it can be concluded that evaluating snacking consumption in isolation as a risk indicator for OrNe tendencies is inadequate. More frequent snacking may reflect a planned, balanced, and metabolically regulated eating approach in some individuals; in other cases, it may become part of a structured, controlled eating pattern based on strict rules. The observation of lower OrNe scores, especially in the group consuming two snacks, suggests that a regular but flexible meal structure may play a protective role. Conversely, the association between very frequent snacking and HeOr is thought to reflect an increased level of interest and planning toward nutrition. Therefore, when evaluating snacking behavior within the context of orthorectic tendencies, it is important to consider not only frequency but also qualitative characteristics such as motivation, level of rigidity, and psychological contributors.

Participants assessed based on their dietary habits revealed that individuals adhering to a diet had significantly elevated mean HeOr scores in comparison to those who skipped a diet. In contrast, people who did not consistently adhere to a diet showed lower OrNe scores. Similar findings were also reported in a study conducted by Styk et al. (60) in a Polish sample. The researchers reported that individuals who practiced restrictive dieting had higher scores for both OrNe and HeOr. The literature generally emphasizes that orthorectic characteristics are associated with increasing dietary restriction (38, 45). Indeed, it has been shown that orthorectic symptoms increase with the intensification of behavioral restrictions, and that orthorectic tendencies are higher in individuals who diet (61). These findings suggest that dieting behavior may overlap with HeOr tendencies; however, it may also be a potential risk indicator for OrNe. Dieting practices, especially in psychologically vulnerable individuals, can develop into obsessive and rigid behavioral patterns over time and therefore require careful evaluation (45). The widespread use of restrictive diets has often been reported to be associated with body dissatisfaction (62). This indicates that dieting behaviors may be related not only to health motivations but also to body image concerns. Furthermore, it has been emphasized that dieting becoming more rigid and compulsive over time may pave the way for the development of eating disorders (63). Additionally, detailed analyses considering the reason for the diet (e.g., medical necessity or body image concerns), its frequency, and form can contribute to a more accurate assessment of orthorectic tendencies. Indeed, one study found a correlation between diet style (vegan, vegetarian, mixed) and frequency of dieting experiences with OrNe; higher OrNe tendencies were observed particularly in individuals following a vegan diet and experiencing dieting more frequently (61). In this context, dietary behavior can reflect a motivation for health-focused eating on the one hand, while on the other hand it can be an indicator of a strict dietary approach. This distinction is important from a public health and clinical perspective because strict and prescriptive dietary patterns are thought to pose a risk for the development of OrNe in some individuals. Therefore, a more comprehensive assessment of dietary behavior seems important for taking necessary preventive measures to avoid future health problems.

We found that supplement users have considerably higher mean HeOr scores than non-users. Our analyses revealed that individuals who regularly use supplements have significantly higher mean HeOr scores compared to those who do not. However, there was no significant difference in OrNe scores based on supplement use. Previous literature has reported conflicting results on this subject. For example, Peres et al. (50), in their research on a Brazilian adult sample, reported that HeOr was more common among those who used dietary supplements (60), in a Polish sample, determined that individuals using supplements had higher OrNe and HeOr scores, but the strength of this effect was higher for HeOr. In another study conducted to determine the prevalence of OrNe, dietary supplement use was found to be associated with higher OrNe scores (64). Another study conducted with university students showed that university students were more likely to develop orthorexia if they were on a diet and used supplements (65). In their study investigating health behaviors associated with OrNe, Oberle et al. (66) reported that the group exhibiting OrNe symptoms used nutritional supplements more frequently than the group with a normal diet. According to these findings, supplement use appears to be associated with increased health awareness and interest in nutrition. However, considering that OrNe is associated with the pursuit of “perfect health” it is thought that this health effort may become rigid, rule-bound, and compulsive over time. This indicates that behaviors that begin with motivation for healthy living may, in some individuals, exceed functional limits and transform into a clinically risky pattern. Therefore, while supplement use alone should not be considered a pathological indicator, it should be carefully considered, especially when accompanied by strict dietary rules, intense health anxiety, and psychological fragility. In this context, it is recommended that experts be sensitive to the potential for health-focused behaviors to evolve into an obsessive and dysfunctional structure over time.

Individuals who spent their entire monthly income on food were found to have higher scores for HeOr compared to those who allocated 1/4, 1/3, and 1/2 of their income to food. Conversely, individuals who did not spend their entire income on food had significantly lower scores for orthorexia nervosa compared to those who did. No research examining the relationship between these variables and orthorectic tendencies was found in the literature. This suggests that the economic resources allocated to food may have different meanings in terms of HeOr and OrNe tendencies.

One of the most conceptually important findings of this study concerns the positive association between AFSS food-insecurity severity scores and both orthorexia dimensions, particularly OrNe. This finding should not be interpreted as an association between orthorexia and food insecurity status, nor as evidence that orthorectic tendencies increase with food security. Rather, it suggests that individuals reporting greater food-insecurity severity may also show stronger health-oriented or rigid food-related concerns. This interpretation is consistent with the broader eating-disorder literature, in which food insecurity has been linked to heightened food preoccupation, disrupted dietary regulation, emotional distress, and maladaptive eating behaviors (67, 68). In the context of orthorexia, food insecurity may operate through a scarcity–control pathway: uncertainty about access to adequate and desirable food may intensify vigilance around food quality, increase cognitive investment in dietary rules, and strengthen the desire to control eating in an otherwise unstable food environment. This mechanism may be particularly relevant for OrNe, where food-related control becomes rigid and psychologically burdensome (67, 69). However, because this study is cross-sectional, the association should be interpreted cautiously and not causally. Future longitudinal research should examine whether food insecurity precedes orthorectic symptoms, whether orthorectic rigidity emerges as a coping response to food-access uncertainty, or whether both are shaped by shared vulnerabilities such as anxiety, dietary restraint, and perceived threat.

Regional analysis revealed that individuals residing in Central Anatolia and Eastern Anatolia exhibited markedly higher average HeOr scores than their counterparts in the Mediterranean region. No substantial variation was observed in OrNe scores based on the regional variable. Regional disparities should be assessed in conjunction with factors such as educational attainment, socioeconomic composition, and cultural dietary practices, rather than just geographical location. Research in Turkey indicates that educational attainment and wealth levels considerably influence food selection criteria, label reading habits, and health-focused choices (70–72). Therefore, considering the socio-cultural and economic context is important in interpreting regional differences. A study showing that food choice criteria differ between regions in Turkey reported that individuals living in Eastern Anatolia are more “careful” when choosing food, while this selectivity may be relatively lower in the Mediterranean region (73). This result is thought to provide a consistent basis with HeOr, which represents “health-oriented, planned, selective” eating attitudes. According to the Turkish Statistical Institute’s (TÜİK) Household Regional Consumption Expenditure (2024) survey, the share of expenditure on food and non-alcoholic beverages is higher in the Eastern/Central regions; similarly, the share of expenditure allocated to alcoholic beverages is lower in the Eastern regions (74). These results show that nutritional behaviors should be evaluated together with regional economic and lifestyle dynamics. Turkey-focused review studies showing differences in eating and drinking habits (local products, home cooking practices, cooking methods, etc.) across regions also emphasize the strong divergence of regional nutritional cultures (75, 76). In this context, we believe that higher HeOr scores in Central and Eastern Anatolia may be related to social norms supporting motivation for healthy living and regular/measured eating practices. In addition, findings in the literature regarding the relationship between urbanization and regional settlement characteristics and eating disorders are inconsistent. van Son et al. (77) reported that urban life may be a potential environmental risk factor for bulimia nervosa; however, Hay and Mitchison (78) emphasized that living in a large metropolis alone is not a strong predictor of developing an eating disorder. Furthermore, a study that found no significant difference in the prevalence of eating disorders among autonomous communities (79) suggests that these disorders may be related to broader shared biological, psychological, and cultural processes rather than regional context. In this context, the absence of regional differences in OrNe scores in our study indicates that OrNe may be more related to region-independent processes such as individual psychological vulnerabilities, cognitive rigidity, and personality traits. In future studies, it is suggested that socioeconomic level, urban–rural settlement, and nutritional motivations (health, weight control, body image, etc.) be modeled together to more clearly differentiate regional effects.

Physically active people have higher HeOr scores than inactive people, according to our research. These data imply that physical exercise may be linked to functional health motivation rather than pathological eating. However, physical activity status did not significantly affect OrNe scores, suggesting that exercise behavior alone cannot cause OrNe. Research links regular physical exercise, particularly professional or extreme sports participation, to greater OrNe levels (38, 63, 80, 81).

In their study of physically active adults of various ages, Brodock et al. (82) found that general physical activity was associated with lower OrNe scores, while more aerobic activity, especially regular strength training, was associated with both HeOr and OrNe scores. In another study of a large sample of 18-59-year-olds, increased activity levels were linked to OrNe and HeOr. In a diverse age group, Peres et al. (50) found that exercise was linked to HeOr and OrNe. Methodological variables including sample age, gender ratio, cultural background, self-report or objective physical activity assessment, and professional or recreational sports participation may explain these variations. The failure to consider physical activity motive (health-, performance-, or appearance-oriented) may explain the disparities between studies. The current findings suggest that evaluating the relationship between physical activity and OrNe tendencies in a one-dimensional way is less explanatory than considering it in terms of exercise type, function, and motivation. Therefore, consider findings from a physically active sample with caution as normative values for the greater population. These differences may stem from methodological variables such as the age distribution of the samples, gender ratio, cultural context, whether physical activity is assessed through self-report or more objective measurements, and whether participants engage in sports professionally or recreationally. In particular, the failure to consider motivation for physical activity (such as whether it is health-oriented, performance-oriented, or appearance-oriented) may be a significant factor explaining the differences in findings between studies. In this context, the current findings suggest that instead of evaluating the relationship between physical activity and orthorectic tendencies in a one-dimensional way, it would be more explanatory to consider it within the framework of the type of exercise, its function, and the determining role of motivation. Therefore, caution is needed when interpreting data obtained from a physically active sample as normative values for the general population.

Like our physical activity study, HeOr and OrNe were positively correlated with eating attitudes and behaviors. We found that sustainable and healthy eating, adult food security, food label reading attitudes, and food selection behavior positively linked with HeOr and OrNe. We also found that both orthorectic tendencies inversely linked with ultra-processed food avoidance. Note that this correlation was weak, especially with OrNe. Until recently, research on healthy and disordered eating behaviors was inconsistent due to insufficient measuring tools (61, 83). Recent research suggests that orthorectic tendencies are not wholly unhealthy. High health sensitivity may be a beginning point for orthorectic orientation.

Beyond these demographic and lifestyle characteristics, the regression analyses suggest that the strongest determinants of orthorectic tendencies are not demographic factors themselves but rather food-related motivations, food security, and social environmental influences. These findings shift the focus from identifying who is more likely to exhibit orthorectic tendencies to understanding the psychological and environmental mechanisms through which such tendencies develop and are maintained.

Among all food-choice motives, weight-control motivation emerged as one of the strongest predictors of orthorexia nervosa. This finding is particularly important because it challenges the common assumption that orthorexia nervosa is exclusively driven by health concerns. Recent studies have demonstrated that pathological orthorexia frequently overlaps with body-image concerns, perfectionism, and restrictive eating patterns typically observed in other eating disorders (8, 42). The positive contribution of weight-control motives observed in the present study supports the view that orthorexia nervosa may involve a concealed focus on body regulation and self-control, even when individuals justify their behaviors through health-related narratives. Consequently, health-oriented discourse may function not only as a nutritional goal but also as a socially acceptable framework through which rigid weight-control behaviors are expressed.

Food insecurity is commonly linked to disordered eating through scarcity-related stress, uncertainty in food access, disrupted meal patterns, compensatory eating, and heightened cognitive preoccupation with food. In this context, the positive association observed in our study may suggest a “scarcity–control” pathway: when access to adequate and desirable food becomes uncertain, some individuals may respond not only with dysregulated intake but also with intensified food vigilance, selective eating rules, moralized food choices, and attempts to impose control over an unstable food environment. Recent literature views orthorexia nervosa as a control-oriented disorder characterized by perfectionism, compulsivity, moralization of dietary decisions, and cognitive rigidity (2, 69, 84). This interpretation is especially relevant for OrNe, which has rigid and compulsive food quality regulation, but it may also explain why HeOr increased with AFSS scores because health-oriented food concerns may become more salient when food access, quality, or nutritional adequacy are threatened. As a cross-sectional study, this link cannot be taken as causative. Instead, it suggests food insecurity may structure adaptive and maladaptive health-oriented eating practices. Future longitudinal studies should examine whether food insecurity precedes orthorectic symptoms, whether orthorectic rigidity develops as a coping response to food-access uncertainty, or whether psychological vulnerabilities like anxiety, perceived threat, perfectionism, or dietary restraint shape both.

The pattern was mostly preserved in multivariate studies. The statistically significant regression models and the fact that the variables analyzed explained 48% of the variance at the HeOr level and 41% at the OrNe level imply that behavioral and environmental factors may be linked to orthorectic inclinations. In particular, the HeOr model’s important contributions of sustainable and healthy eating behaviors, food security, food label reading attitudes, and food choice behavior reflect a knowledge-based and regulatory health orientation. The regression analysis showed that sustainable and healthy eating behaviors were the strongest predictors of HeOr, supporting the assumption that this dimension is primarily related to healthy eating behavior intensification.

However, ultra-processed food avoidance and economic drivers in the OrNe model show more rigid and selective patterns in this dimension. However, sustainable eating behaviors and social variables in both models suggest that health sensitivity may underlie both orthorectic features. Flexible and functional health sensitivity is connected with HeOr, while rigid, controlling, and compulsive is associated with OrNe. The literature defines HeOr as a conscientious, functional approach to healthy eating, while OrNe is a strict, compulsive, and dysfunctional form (11, 68). Cognitive flexibility, perfectionism, control, and self-esteem regulation may separate these strategies. Other studies have linked orthorectic tendencies to psychological processes (85, 86). We believe that the psychological processes that process this health-focused orientation may determine whether the outcome is HeOr or OrNe.

In summary, based on the available data, “health-oriented attitudes and behaviors” can be positioned as a key antecedent variable in the model proposed within the scope of this research. This orientation, when combined with cognitive flexibility, self-compassion, and functional self-regulation, can transform into a pattern associated with HeOr. Conversely, the same orientation can progress toward OrNe when combined with processes such as cognitive rigidity, perfectionism, need for control, and the linking of self-worth to external standards. Therefore, the fundamental element distinguishing orthorectic tendencies is not the content of the behavior, but rather the underlying cognitive and motivational structure of the behavior. In this context, process-based approaches that focus not only on the presence of the behavior but also on its function, level of flexibility, and the individual’s self-regulation capacity may be more explanatory in the evaluation of orthorexia. We believe that future research testing the moderating role of variables such as cognitive flexibility, perfectionism, body image motivation, and exercise motivation could more clearly reveal the mechanisms of divergence between these two dimensions.

Finally, this study found that the physical, social, and economic sub-dimensions of the obesogenic environment were positively correlated with both HeOr and OrNe, while its cultural determinants were negatively correlated with both orthorectic tendencies. Although the relationship between obesogenic environment and obesity and unhealthy eating behaviors has been extensively examined in the existing literature (87–90), no study has been found that directly addresses the relationship between these environmental factors and orthorectic tendencies. In this respect, the findings suggest that the obesogenic environment can create not only for excessive energy intake but also for overly controlled and rigid eating patterns. The correlation findings, supported by multivariate analyses, reveal a more consistent pattern in the relationship between obesogenic environmental dimensions and orthorectic tendencies. Obesogenic environment; Dietary factors include easy access to energy-dense foods, high levels of unhealthy eating habits and fast food, limited physical activity areas, and socioeconomic disadvantage (91, 92). It is suggested that such environmental conditions can increase individuals’ perception of health threats and trigger compensatory control strategies in response. Ecological models emphasize that individual eating behaviors are sensitive to the environmental context (93). Similarly, the social determinants framework reveals that low socioeconomic conditions and stress shape eating behaviors (94). In this context, it can be considered that continuous exposure to an unhealthy food environment may lead some individuals to develop a counter-response in the form of a “healthy eating orientation.” However, it is thought that this orientation may remain flexible and functional in some individuals HeOr, while in others it may transform into a rigid and dysfunctional form OrNe. The findings indicate that environmental pressure can increase motivation for healthy eating, but this motivation can be organized differently depending on individual and psychological characteristics. On the other hand, the negative relationship between cultural determinants and orthorectic tendencies is noteworthy. Cultural dietary norms, traditional eating practices, and collective food culture can play a limiting role in individual tendencies toward strict control. It is known that the cultural context normatively regulates eating behaviors and assigns social meaning to nutrition (93, 95). In environments with strong cultural dietary norms, eating behavior can be organized as a collective sharing practice rather than an area of individual performance and control. This can weaken the individual, rule-bound, and strict eating tendencies seen in orthorexia. Therefore, an increase in cultural determinants can serve a protective function for orthorectic tendencies. These findings reveal that the effect of an obesogenic environment is not one-sided, but presents a complex structure that can encourage not only excessive consumption but also excessive control.

The strongest environmental predictor of orthorexia nervosa in the present study was the social dimension of the obesogenic environment. This finding highlights the importance of considering orthorexia within a broader sociocultural context. Contemporary food environments are characterized by constant exposure to nutrition-related information, wellness culture, social media content, and normative pressures promoting dietary self-optimization. Recent evidence suggests that social comparison, exposure to idealized dietary practices, and perceived pressure to eat “correctly” may reinforce orthorexic tendencies (5, 49). The present results indicate that social environmental influences may contribute to orthorexia nervosa not by encouraging unhealthy eating, but paradoxically by promoting excessive vigilance, perfectionism, and dietary control. This interpretation supports ecological models of eating behavior, which emphasize that food-related psychopathology develops through interactions between individual vulnerabilities and environmental influences.

Taken together, the findings suggest that HeOR and OrNE should not be viewed as opposite ends of a single continuum. Rather, they appear to represent distinct expressions of a shared interest in food and health that diverge through different motivational and environmental pathways. However, the term “pathway” is used here in a conceptual rather than causal sense; the present cross-sectional data can identify patterns of co-occurrence but cannot determine whether these motivational and environmental factors precede, follow, or mutually reinforce orthorectic tendencies. Healthy orthorexia was primarily associated with adaptive health-oriented motives, nutritional awareness, and sustainable eating behaviors. In contrast, orthorexia nervosa was more strongly linked to weight-control concerns, moralized food beliefs, social environmental pressures, and rigid forms of dietary self-regulation. These findings contribute to the growing body of evidence supporting the conceptual distinction between healthy and pathological orthorexia and suggest that future prevention strategies should focus not only on what individuals eat but also on why they eat and how social environments shape food-related identities and behaviors. The clinical and theoretical boundary between HeOr and OrNe may therefore lie less in the intensity of healthy-eating interest itself than in whether that interest is accompanied by flexibility and well-being or by rigidity, distress, and impairment.

## 5 Strengths and limitations

This study has several important strengths. First, we examined orthorexia using a contemporary, two-dimensional framework, employing the Teruel Orthorexia Scale to differentiate HeOr from orthorexia nervosa. This aimed to eliminate the handicaps of older, widely criticized instruments with poor psychometric performance and incompatibility with health-conscious eating behaviors. This distinction significantly increased the conceptual clarity of the findings and allowed for a more nuanced interpretation of how similar health-focused behaviors can be categorized into functional and dysfunctional patterns.

Second, the study comprises a relatively large adult sample (*n* = 1,015) spanning a wide age range (18–65 years), enhancing statistical power and allowing for the simultaneous assessment of multiple sociodemographic, behavioral, and environmental factors.

Thirdly, the analytical strategy is stronger than that of average cross-sectional orthorexia studies: linear regression assumptions were controlled, outliers were systematically screened, and multivariate models were used to examine the independent variables of the sub-dimensions of sustainable and healthy eating behaviors, ultra-processed food consumption, AFSS food-insecurity severity score, food label reading attitudes, food choice, and obesogenic environment. The explained variance is also significant, especially for HeOr (R^2^ = 0.40), indicating that the model captures a significant portion of the observed pattern.

Another important strength is the breadth of the explanatory framework. Most studies on orthorexia still focus narrowly on psychological characteristics, body image, or exercise; our research, however, expands the field by integrating AFSS food-insecurity severity score and obesity-causing environmental dimensions, which have not been sufficiently examined in orthorexia research. This adds a real novelty to our research and demonstrates its significant contribution to the literature.

The limitation is that the cross-sectional design prevents causal inferences. Our regression model identifies statistical relationships but does not reveal temporal relationships. Another statistical limitation is that no formal correction for multiple comparisons was applied to the exploratory *t*-tests, ANOVA analyses, or bivariate correlations. The correlations identified in this study should be regarded as a cross-sectional explanatory pattern rather than as proof of causal relationships. While the regression models found variables that statistically accounted for variance in HeOr and OrNe, the temporal direction of these connections remains uncertain. Consequently, the current findings should be seen as revealing high-priority correlates and generating hypotheses for future longitudinal, ecological momentary assessment, and intervention studies rather than establishing causal determinants. The study sample includes people from varied demographic and lifestyle backgrounds, but it was not probability-based, the conclusions should not be applied to all adults living in Turkiye.

A second limitation is the reliance on self-reported measures for all baseline constructs, including dietary habits, food insecurity status, physical activity, and environmental characteristics contributing to obesity. This creates a risk of common method variance, recall bias, and social acceptability bias, especially in an area where participants may wish to portray themselves as health-conscious.

Third, while the sample size is robust, representativeness remains limited. The paper describes the completion of face-to-face interviews and voluntary participation, but does not provide a population-based sampling framework; therefore, caution should be exercised before generalizing the findings to all adults in Türkiye.

Fourth, because AFSS was analyzed as a continuous food-insecurity severity score, the findings should be interpreted in relation to increasing food insecurity rather than increasing food security; future studies should examine categorical food-security status and test whether the association differs across food-secure, low-food-security, and very-low-food-security groups.

Fifth although conceptual progress has been made in separating HeOr and OrNe, the study did not address psychological process variables.

Finally, limitation is that the study modeled HeOr and OrNe as continuous outcomes and did not group individuals into HeOr-dominant, OrNe-dominant, high-both, or low-both profiles. The positive link between HeOr and OrNe shows considerable overlap, but this design cannot differentiate which individuals are predominantly characterized by adaptive healthy-eating motivation vs. maladaptive orthorectic rigidity. To understand how people go from flexible health-oriented eating to strict and damaging orthorexic patterns, future research should use person-centered methodologies like latent profile analysis or longitudinal trajectory models with psychological and clinical impairment measurements.

## 6 Conclusion

This study concludes that HeOr and OrNe are linked to unique yet somewhat overlapping patterns of dietary behavior, severity of food insecurity, reasons for food choices, and perceived obesogenic environmental influences. These findings underscore the necessity of assessing orthorectic inclinations within a multilevel framework that amalgamates individual dietary intentions with contextual food-environment variables, acknowledging that the cross-sectional design precludes causal or temporal inferences. We believe that using longitudinal and experimental designs in future research could more clearly explain the direction of these relationships. In addition, we believe that testing whether the differentiation of orthorectic tendencies based on a shared health orientation is related to perceived threat and protective behavior motivations defined in health belief models could be valuable for future research. In particular, examining the extent to which perceived risk, benefit, and obstacle components explain the divergence between HeOr and OrNe using longitudinal and experimental designs could yield clearer results. The significance of the current study resides not in establishing causality, but in delineating a multilayer array of correlates that can inform future longitudinal and mechanistic investigations into the conditions under which health-oriented eating is adaptive vs. when it becomes inflexible, compulsive, and clinically pertinent.

## Data Availability

The original contributions presented in the study are included in the article. Further inquiries can be directed to the corresponding authors.
